# A review of sperm cryopreservation in the domestic dog and cat: part I, from science to clinic

**DOI:** 10.1186/s13028-025-00845-2

**Published:** 2025-12-11

**Authors:** Eva Axnér

**Affiliations:** Department of Clinical Sciences, Swedish University of Agricultural Sciences, P.O. Box 7054, 750 07 Uppsala, SE Sweden

**Keywords:** Canine, Feline, Freezing, Reproduction, Semen, Spermatozoa

## Abstract

Semen cryopreservation is widely applied in the breeding of several domestic animal species. In the domestic dog, artificial insemination with cryopreserved semen is now routinely performed, whereas in the domestic cat the technique is more challenging. Achieving acceptable pregnancy rates requires high post-thaw semen quality. Unfortunately, sperm cells are susceptible to damage caused by temperature reduction. At temperatures below − 130 °C harmful reactions that can damage spermatozoa are essentially halted. Therefore, spermatozoa can be stored in liquid nitrogen at -196 °C for virtually unlimited periods, enabling the transport of genetic material across both time and space. To reach such low temperatures, however, sperm cells must undergo detrimental changes in temperature. Sperm samples are therefore diluted in buffered extenders containing cryoprotective agents that reduce cold shock and freezing induced damages. Despite these measures, freezing and thawing inevitably cause cell injuries, resulting in reduced longevity compared with freshly ejaculated spermatozoa. Therefore, intrauterine insemination and accurate prediction of ovulation are required to achieve acceptable pregnancy results. Protocols for cryopreservation of dog and cat spermatozoa are often adapted from those developed for other species, in which semen preservation is more established. However, sensitivity to cold shock and freezing varies both between species and among individuals, largely due to differences in sperm cell membrane composition. Moreover, spermatozoa from different species may exhibit varying degrees of sensitivity to potentially toxic effects of ingredients in semen extenders. Thus, protocols must be tailored to each species. Understanding mechanisms of cryo-induced cell damage requires a fundamental understanding of how cells are affected by low temperatures. Much of the research on basic cryobiology was conducted decades ago, yet improvements in cryopreservation protocols are still in progress, often driven by empirical studies, comparing alternative strategies. The aim of this review is to synthesize current knowledge on canine and feline semen freezing, placing recent findings in the context of historical research. Several breakthroughs in cell cryobiology have been successfully applied in these species and are still commonly used. Such examples are the basic Tris-buffer, and the use of egg yolk and glycerol in freezing extenders. Future developments may include alternatives to antibiotics and replacement of egg yolk with non-biological alternatives.

## Background

Semen cryopreservation is widely applied in the breeding of several domestic animal species. Although, not as extensively used, as in for example cattle, it is a valuable tool also in dog breeding. The first birth of a puppies following artificial insemination (AI) with cryopreserved semen was reported 1969 [1]. Since then, advances in cryopreservation and insemination techniques, have enabled the routine use of frozen-thawed canine semen, achieving acceptable to good pregnancy rates [2]. Intrauterine insemination with frozen-thawed dog semen typically yields pregnancy rates of around 70% or higher [2, 3]. Achieving such outcomes requires high post-thaw semen quality, intrauterine deposition of the semen, and a reproductively healthy bitch. Due to the reduced longevity of frozen-thawed spermatozoa, precise timing of insemination relative to ovulation is also essential to optimize pregnancy results [4]. Semen from the domestic cat can also be successfully cryopreserved [5]. The first successful AI with frozen-thawed feline semen was reported 1978 [6], however, AI with frozen-thawed semen in cats has not yet become a routine clinical procedure. The challenges in this species are greater than in the dog, owing to more complex semen collection, small ejaculate volumes, low sperm counts per ejaculate, and often a high proportion of morphologically abnormal spermatozoa [7, 8]. The aim of this review is to synthesize current knowledge on canine and feline semen cryopreservation and the development of successful protocols. Historical data are included to provide a background to more recent findings, and their relevance to the clinical application of semen cryopreservation in these species. The focus will be on conventional freezing methods, as alternative approaches such as vitrification and freeze-drying while promising, are not yet routinely used in clinical practice and are therefore outside the scope of this review and will only be mentioned briefly.

## Search strategy

This narrative overview is based on a search in PubMed (https://pubmed.ncbi.nlm.nih.gov/) and Web of Science (https://www.webofscience.com/wos/alldb/basic-search) using the terms “canine, cold shock, cryocapacitation, cryopreservation, equilibration, extenders, feline, freezing, semen, sperm, spermatozoa.” In addition, the author’s own archive on ”Endnote TM^20^ (Clarivate analytics)” was used as a source. References dating between 1940 until current have been included to cover the historical development of sperm cryopreservation of relevance for the canine and feline species.

## Review

### Cold shock

In canine and feline semen cryopreservation, the semen sample is typically cooled to 4–5 °C before freezing. Spermatozoa are, however, susceptible to damage caused by a temperature reduction, a phenomenon known as cold shock. Cold shock results in loss of sperm function and membrane integrity. Bowing of the sperm mid piece, bent sperm tails, and loss of motility may be visible signs of cold shock [9]. Most of the cold-shock related damage originates from alterations in the plasma membrane. As temperature decreases, cell membrane lipids undergo phase transitions from liquid to gel phase [10]. This reorganisation increases permeability leading to leakage of potassium and uptake of Ca^2+^ [10, 11]. Rapid cooling is generally more harmful than cooling at a slower rate [9]. Sensitivity to cold shock varies between species and individuals, depending largely on the composition of the cell membrane. The higher the ratio of unsaturated/saturated fatty acids and the lower the cholesterol content in the sperm membrane, the more susceptible the cell is to cold shock [12]. For example, canine spermatozoa are less sensitive to low temperatures than ram and bull spermatozoa [13]. Feline spermatozoa appear to have an intermediate sensitivity, greater than human spermatozoa but lower than ram and bull, similar to the dog [14]. Recent findings indicate that cat spermatozoa contain a low unsaturated/saturated fatty acids ratio [15], which may explain their relative resistance to cold shock [12]. Experimental data support that cooling rates of 0.125–3 °C/minutes (min) are well tolerated by cat spermatozoa [16, 17], while rapid (4 °C/min), or ultrarapid (14 °C/min) cooling causes acrosomal damage [18]. In dogs, cooling semen to 5 °C at either 0.2 °C/min or 2.25 °C/min, showed not significant differences in sperm quality parameters [19]. In species, such as bulls and boars, it has since long been known that equilibration after semen collection increases resistance to freezing injuries. During equilibration, the diluted semen sample is cooled and incubated before freezing. This was initially thought to allow time for glycerol penetration, but glycerol permeates rapidly and does not require prolonged equilibration [9, 20]. In canine cryopreservation, equilibration times at 4–5 °C vary between protocols. Early studies applied a 3-h equilibration [1, 21], while later work showed no difference between 1, 2, and 3 h equilibration [22]. In the widely used Uppsala two-step method, semen is cooled for 75–90 min [23, 24]. The role of equilibration time is even less systematically explored in the feline where protocols are often adapted from canine semen freezing [5]. In conclusion, both canine and feline spermatozoa appear relatively tolerant to the cooling rates commonly used in semen cryopreservation, suggesting that the cooling step itself is not a major determinant of post-thaw quality. However, excessively rapid or ultrarapid cooling should be avoided. Controlled, gradual cooling can be achieved using an automatic freezer, a cold-handling cabinet, or by placing diluted semen in a water jacket at room temperature before refrigeration [25]. While cooling might not be a very critical step in feline and canine sperm cryopreservation, freezing will induce severe damages on the spermatozoa.

### Freezing injuries

Long-term preservation of spermatozoa requires temperatures below − 130 °C where liquid water no longer exists and ice crystal formation cannot occur [26, 27]. Spermatozoa can therefore be stored for an almost unlimited time in liquid nitrogen (LN_2_) at -196 °C. The only processes that can harm spermatozoa during LN_2_-storage are events caused by back-ground radiation and cosmic rays, which eventually lead to DNA damage [26]. Thus, semen cryopreservation enables the transport of genetic material across both time and space.

**Fig. 1:**
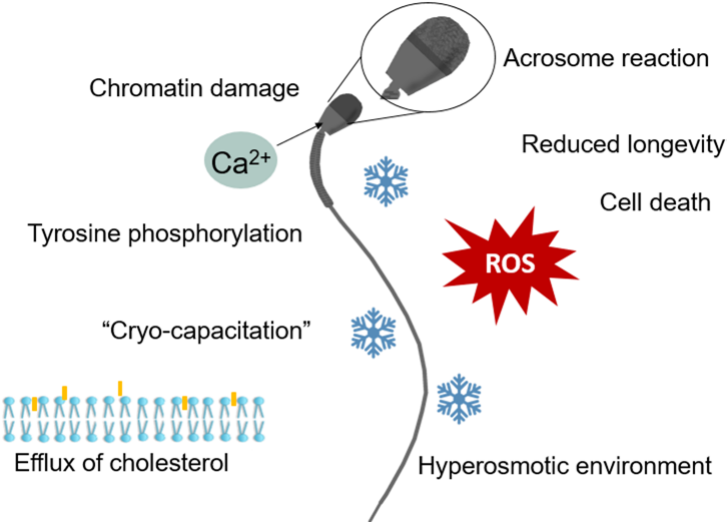
Cryo-induced sperm damages. Formation of ice crystals cause the extracellular environment to become hyperosmotic, causing osmotic stress. Intracellular ice-crystals, caused by rapid freezing rates, are often lethal. Cryopreservation cause changes similar to capacitation, which reduce longevity of the sperm cells. Reactive oxygen species (ROS) are formed during the semen preservation procedure

However, to reach these protective temperatures, spermatozoa must traverse a lethal temperature zone, approximately between − 15 °C and − 60 °C, where most cryoinjuries occur [26]. Below 0 °C, sperm damages are caused by formation of ice crystals and concentration of solutes in the remaining water fraction (Fig. 1) [11]. At a slow freezing rate, water will leave the cell as ice crystals are formed in the extracellular water. The environment surrounding the sperm cells becomes hyperosmotic, as solutes are concentrated in the remaining water fractions [26, 28]. If the cooling rate is too slow, volume changes caused by dehydration, and toxic effects of increased solute concentrations will harm the sperm cells [28]. Conversely, if the freezing rate is too fast water cannot leave the cell in time, and lethal intracellular ice forms [11, 26]. In practice, the very rapid cooling rates required for intracellular ice formation are not typically applied to semen freezing. Thus, cryoinjury is most often associated with osmotic stress and solute effects during slow cooling [29]. An optimal cooling rate for sperm cells has been stated to be between 10 °C/min and 80 °C/min [11]. This corresponds well with empirical results from studies on canine semen freezing. Moderate rates of 20–50 °C/min through the critical zone yield the best outcomes [30–33], while very slow rates (≤ 10 °C/min), or very fast rates ≥ 75 °C/min) are detrimental [31, 33]. For feline spermatozoa, a rate of 10 °C/min down to − 80 °C produced superior results compared with rapid pellet freezing on dry ice [34]. Another study suggested that 3.85 °C/min yielded better outcomes than 9 °C/min or faster, although temperatures were measured in the freezing device rather than within straws [35]. Spermatozoa are usually frozen in LN_2_ vapour. In the classical box method, described by Andersen [21, 36], semen is packaged in 0.5 mL straws, placed on a rack 4 cm above LN_2_ for 8 min, before plunging the straws into LN_2_. Variations of this method have been described, involving adjustments in vapour exposure time, straw volume (0.25 vs. 0.5 mL), and height above the LN₂ surface [30, 33, 37–39]. Alternative approaches include gradually lowering the straws into a LN_2_-tank [30, 40], or using a programmable freezers to regulate cooling [32]. Another method is pellet freezing where semen is dropped directly onto a block of dry ice before transfer to LN₂ [1, 41] (see below about packaging in pellets). Freezing will cause subtle membrane changes, that shorten the longevity of spermatozoa.

#### Cryo-capacitation

The cell membranes of frozen-thawed spermatozoa exhibit increased fluidity and reduced stability compared with those of fresh spermatozoa [9]. Membrane reorganization, accompanied by increased permeability to Ca^2+^ [9], and cholesterol efflux [42], resembles the physiological changes that occur during sperm capacitation. These alterations contributes to the reduced longevity of cryopreserved spermatozoa [9]. In canine spermatozoa, this phenomenon has been confirmed by a lower proportion of uncapacitated spermatozoa, as assessed by the chlortetracycline (CTC) assay in frozen-thawed semen samples [43], along with elevated intracellular Ca^2+^ concentrations [44].

#### Oxidative stress

**Fig. 2:**
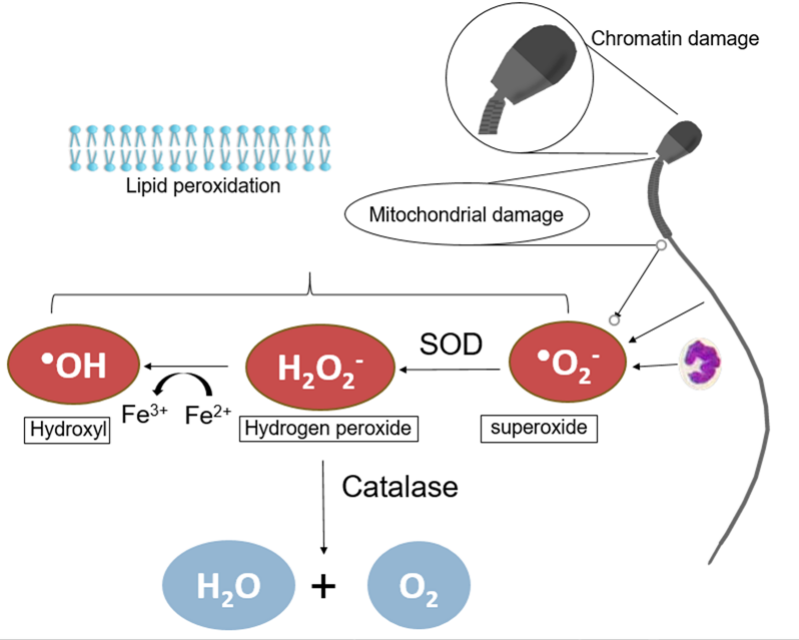
Sperm damages caused by oxidative stress. Reactive oxygen species (ROS) are produced by spermatozoa and leukocytes in the semen. Superoxide dismutase (SOD) removes superoxide generating hydrogen peroxide, which can be transformed to harmless water and oxygen by the action of catalase. In the presences of ferrous ions, hydrogen peroxide can form hydroxyl radicals. ROS can cause lipid radicals that attack the sperm membranes, and self-propagate in a chain reaction

During sperm conservation, reactive oxygen species (ROS) are generated. Excessive ROS attack sperm membranes, induce lipid peroxidation and thereby impair sperm quality (Fig. 2) [11, 45, 46]. Sperm membranes with a relatively higher content of polyunsaturated fatty acids (PUFA) are particularly vulnerable to oxidative damage [47]. However, ROS are not only detrimental: low concentrations are necessary for sperm function and fertilization [48]. Thus, there must be a balance between ROS production and inactivation. Sources of ROS include leukocytes, as well as dead and immature spermatozoa [49, 50]. Endogenous antioxidants present in seminal plasma play a crucial role in counteracting ROS and maintaining a balance [50].

#### Individual differences in freezability

In dogs and cats, as in other species, sperm freezability varies between individuals. The underlying causes for this variability remain insufficiently explored. A high seminal plasma cholesterol concentration has been associated with a better post-thaw sperm quality in cats and dogs [51, 52]. In cats, higher seminal triacylglyceride concentration has also been linked to better sperm cryosurvival [51]. There are contradictory results on the importance of sperm morphology on feline sperm cryotolerance. Cats with >40% morphologically abnormal spermatozoa were found to be more susceptible to acrosomal damage following rapid or ultrarapid cooling [18], and more sensitive to hypotonic stress [53], compared with cats with >60% morphologically normal spermatozoa. However, the predictive value of sperm osmotic tests, and markers of sperm membrane stability, was found to be weak in a more recent study [54]. Furthermore, the percentage of abnormal spermatozoa was not associated with freezability in either cat epididymal [5], or ejaculated spermatozoa [51].

### Semen extenders

Without semen extenders, spermatozoa would not survive the freezing process. In the earliest reports of successful AI with frozen-thawed semen in the dog and cat, an 11%-lactose extender was used [1, 6]. Early experiments demonstrated that a controlled pH and a buffer system were essential for successful semen preservation at low temperatures [55]. Today, most non-commercial extenders used for dog semen cryopreservation are based on modification of the Tris-citrate-egg yolk extender (TEY), originally developed for bull semen (Table 1) [21, 24, 40, 56, 57]. Tris (tris(hydroxymethyl)aminomethane) is buffered with citric acid to achieve a suitable pH. A TES-Tris (NTris(Hydroxymethyl)methyl-2-Amino-Ethanesulfonic Acid (TES)-Tris buffer) is also commonly used for feline extenders [17, 58]. In the unfractionated ejaculate, dog semen has a pH of approximately 6.4 [59] and an osmolarity of ∼316 mOsm [60]. Cat semen varies between 320 and 345 mOsm, with a pH of ∼6.6 [61, 62]. For canine, sperm motility is best maintained at a pH of 6.6 compared with 5.9 or 7.3, and with an osmolarity between 280 and 355 mOsm [63]. Similarly, an osmolarity between 292 and 325 mOsm was optimal for feline spermatozoa [62], suggesting that extenders should closely match the physiological pH and osmolarity of the ejaculate. However, these values apply only before glycerol is added, since glycerol substantially increases extender osmolarity (Table 1). Glycerol, however, permeates the cells over time and thus does not contribute to extracellular osmotic pressure. Addition and removal of glycerol may subject the spermatozoa to an osmotic stress before an equilibrium is achieved [9] (see more below about glycerol).

**Table 1:** Examples of Tris-based extenders based on, and modified from [56]. Ingredients/100 mL

References	Davis et al. [56]	Foote [57]	Thomas et al. [37]		Rota et al. [40]		Linde Forsberg [23]	
			1	2	1	2	1	2
Tris	2.42	2.42 g	2.4 g	2.4 g	2.4 g	2.4 g	3.025 g	3.025 g
Citric acid	1.35 g	1.35 g	1.4 g	1.4 g	1.4 g	1.4 g	1.7 g	1.7 g
Fructose	–	–	–	–	–	–	1.25	1.25
Glucose	–	1 g	0.8 g	0.8 g	0.8 g	0.8 g	–	–
Glycerol	7 g	11 mL	–	6 mL	3 mL	7 mL	3 mL	7 mL
Orvus paste	–	–	0–2 mL	0–2 mL				
Equex STM	–	–	–	–	–	1 mL	–	1 mL
Egg yolk	20 mL	20 mL	20 mL	20 mL	20 mL	20 mL	20 mL	20 mL
Penicillin	5000 IU	10,000 IU	5000 IU	5000 IU	0.06 g	0.06 g	0.06 g	0.06 g
DHS*	5000 µg		–	–	–	–	–	–
Streptomycin	–	–	0.1 g	0.1 g	0.1 g	0.1 g	0.1 g	0.1 g
pH	6.74		–	–	6.53	6.48	6.72	6.74
Osmolarity mOsm	–	–	–	–	740	1370	865	1495

*DHS* dihydrostreptomycin

1) A buffer was prepared with 3.028 g Tris, 1.69 g citric acid and 1.25 g glucose. The buffer was mixed 80:20 with egg yolk to the final concentrations in the table

2) Semen was diluted to 300 × 10^6^ spermatozoa/mL with the first extender and further diluted twice with the second extender with 30 min intervals to give a final concentration of 4% glycerol and 100 × 10^6^ spermatozoa/mL

3) Uppsala Equex-I. A two-step dilution protocol was applied. The second extender was added, in the same volume as the first, after the equilibration period just before filling the straws. The final glycerol concentration is 5%. The osmolarity of the extender without glycerol and Equex STM (used as a thawing medium) was 253 mOsm

4) Uppsala Equex-II. A two-step dilution protocol as in [40] was applied. Instead of adding water to 100 mL as in Davis and Foote and mixing with 80:20 with egg yolk, water is added to 77 and 72 mL. Glycerol, egg yolk and Equex STM paste are thereafter added to 100 mL. The osmolarity of the extender without glycerol and Equex STM (used as a thawing medium) was 324 mOsm

#### Non-penetrating cryoprotective agents

Cryoprotective ingredients (CPAs) are added to semen extenders to protect the cells from damages caused by cooling and freezing. CPAs are classified as non-penetrating or penetrating, depending on whether they remain extracellular or cross the sperm membrane. The most widely used non-penetrating CPAs are egg yolk and sugars. The protective effect of hen’s egg yolk was an early breakthrough in semen preservation [55], mediated primarily by its low-density lipoprotein fraction (LDL) [64]. In the bull, LDLs form complexes with seminal plasma proteins present on sperm membranes, thereby stabilising the sperm membranes by counteracting efflux of cholesterol, and phospholipid removal [65]. Typically, 20% egg yolk is added to canine and feline semen extenders [21, 40, 57, 66, 67]. Its protective effect can be enhanced by detergents such as Sodium Dodecyl Sulphate (SDS, a component in Equex STM or Orvus paste) to the extender [68]. In both dogs and cats, SDS supplementation improves post-thaw sperm quality when combined with egg yolk [5, 37, 40, 69]. In the absence of egg yolk in the extender, SDS is detrimental [68, 70].

Egg yolk is a biological product that unfortunately varies in composition and has a risk of being contaminated with pathogens [71]. Other sources of phospholipids as alternatives to egg yolk have therefore been explored. Replacement of egg yolk with egg yolk plasma (EYP) has shown some promising results [72, 73]. Results are, however, conflicting. When EYP was compared with egg yolk in a TEY-Equex STM Paste freezing extender, egg yolk yielded better results [74]. Early studies demonstrated that the purified LDL fraction of egg yolk had protective effects during sperm cryopreservation in the ram and bull [64]. LDL has also been used successfully for cryopreservation of canine spermatozoa [75]. However, in studies on LDL the total motility in cryopreserved semen frozen with egg yolk seems to be lower (mean values < 50%) [73, 76] than usually achieved with traditional TEY-Equex extenders [30, 40, 74]. Differences in freezing protocols and composition of extenders could also be a reason for differences between studies. A drawback with LDL is that extractions protocols are complicated and not practical for routine clinical application [75].

Soybean lecithins have also been used as alternatives to egg yolk for semen cryopreservation. There are different sources of soybean lecithin with somewhat different composition of phospholipids, why the choice of type of soybean lecithin, and concentration may affect the results [77]. Most of the studies showed superior results for egg yolk compared with soybean lecithins for canine spermatozoa [78–80], while some had similar post thaw semen quality [70, 81]. In the cat, a positive effect of soybean lecithin compared with egg yolk was found [58].

Sugars have several benefits in semen extenders. Sperm cells can metabolise monosaccharides. These sugars will therefore provide energy [82, 83], while for example lactose and raffinose mainly act as cryoprotectants [84]. The cryoprotective effect of sugars is believed to be to increase the unfrozen water fraction, and thereby reduce the extracellullar salt concentration during freezing [85]. Addition of sugars will also contribute to the osmolarity of the buffer. While glucose and fructose are most common (Table 1), other sugars (galactose, maltose, raffinose, sucrose, trehalose, xylose) have been tested for canine and/or feline sperm cryopreservation [1, 6, 86, 87]. Recently, dextran showed promise as an alternative to egg yolk for feline sperm cryopreservation [88].

#### Penetrating cryoprotective agents

The discovery of the positive effects of glycerol was a major breakthrough in cryobiology [89]. Glycerol remains the most used cell penetrating cryoprotectant for dog and cat semen freezing. It reduces extracellular salt concentrations by increasing extracellular fluid volume [26]. However, glycerol can also be cytotoxic [9], requiring an optimal balance between toxic and cryoprotective effects. In both dogs and cats, 5% glycerol generally yields superior outcomes compared with 3% [32, 38]. In cats, 7% glycerol was more detrimental than 5% [38], whereas in dogs, 7% performed better than 5–6% in a recent study [90]. Other penetrating CPAs, including dimethyl sulfoxide, ethylene glycol, propylene glycol, dimethyl formamide, DMSO, and methyl acetamide, have been evaluated in canine and feline sperm cryopreservation, but generally showed no clear advantage over glycerol [22, 90–94].

#### Antibiotics

Because semen collection is not sterile, bacteria from the normal genital microbiota are often present in samples [95]. Bacteria may negatively affect sperm quality. There is also a perceived risk that bacteria may be transmitted to the female during insemination and cause disease. For this reason, antibiotics are routinely added to semen extenders (Table 1). However, the use of subtherapeutic antibiotic concentrations raises concerns regarding antimicrobial resistance [96, 97]. Antibiotics may also have negative effects on the sperm cells [96], and pose hazards for laboratory staff preparing extenders. Alternative approaches include reducing bacterial load by colloid centrifugation [98], or incorporating plant-based extracts with antibacterial properties. Such extracts have shown promising results in semen from various species, but species-specific optimization is required to ensure efficacy without harming spermatozoa [99].

#### Other ingredients in semen extenders

Antioxidants have been incorporated into semen extenders to counteract the deleterious effects of ROS. As with other ingredients in semen extenders, empirical studies are required to identify optimal compounds and concentrations that improve post-thaw sperm quality. In dogs, beneficial effects have been reported with the addition of taurine [100], reservatrol [101], curcumin [102], ascorbic acid [103, 104], glutathione [103], catalase, NAC, and vitamin B16 [104], although results with melatonin remain inconsistent [105, 106]. In cats, positive outcomes have been observed following supplementation with taurine DL-cysteine, Trolox (vitamin E analogue) [107], glutathione peroxidase [108], L-carnitine [109], whereas catalase or superoxide dismutase showed no beneficial effects [108, 110].

Some species naturally produce antifreeze proteins (AFP) that prevents ice crystal formation. These proteins enhance survival at subzero temperatures [111]. AFPs have been added to semen extenders in different species. In cats, AFP I supplementation yielded no consistent improvements in frozen–thawed epididymal sperm quality [112], whereas in dogs, AFP III increased post-thaw semen quality [113].

As mentioned above, cryopreservation causes cholesterol efflux from sperm membranes, increasing membrane fluidity and reducing stability. Supplementation with cholesterol-loaded cyclodextrins has been shown to improve post-thaw motility parameters, and sperm viability in dogs. However, capacitation status remained unchanged, and oocyte binding ability was reduced [41]. Promising results thus suggest that increasing cholesterol in the plasma membrane of canine spermatozoa can improve their freezability [67, 114]. However, it remains to be elucidated if increasing sperm membrane cholesterol has positive effects on fertility, as membrane stabilization may not always be reversible, potentially impairing capacitation and fertilization [29].

### Semen packaging and marking

The most common packaging methods are mini straws (0.25 mL), medium straws (0.5 mL), and pellets stored in cryovials. Semen was frozen in pellet in the first reports on successful of AI with frozen-thawed semen in the dog and cat [1, 6]. Later studies showed that 0.25 mL straws preserve feline sperm quality better than pellets [17, 34], although another study found no significant difference [115]. In dogs, 0.5 mL straws appear superior to 0.25 mL [33], and more practical in clinical settings. In cats, due to small ejaculate volumes and sperm counts, 0.25 mL straws or pellets are generally preferable. However, pellet freezing raises concerns regarding cross-contamination, limited labelling options, and reduced hygiene compared with straws [23, 116, 117]. In the European Union, semen labelling is regulated. Each straw must include: the date of semen collection, species (and subspecies if relevant), donor identification (e.g., microchip number), and establishment of collection (unique registration number of the establishment can be used) [118]. Although not legally required, including donor breed and name is recommended to facilitate correct dose identification.

### One- or two-step dilution

Because glycerol addition imposes osmotic stress on spermatozoa [9], some protocols increase glycerol concentration in two or more steps [5, 30, 37, 40]. In a two-step protocol, the first dilution is performed at room temperature, and the second after cooling, just before freezing. Canine spermatozoa cryopreserved in the Uppsala I extender (Table 1) exhibited better post-thaw quality with a two-step dilution compared with one-step dilution, but only when Equex STM paste was included [30]. This suggests that the benefit may be due to minimizing prolonged SDS exposure rather than reducing glycerol-related osmotic stress [30].

### Dilution rate and removal of seminal plasma

Protocols differ in whether canine ejaculates are diluted by volume-to-volume ratio [21], or according to sperm concentration [37, 119]. Many protocols also include seminal plasma removal by centrifugation prior to extender addition [23, 40]. These differences in protocols will obviously affect the final concentrations of CPA, SDS, and other extender components.

The impact of seminal plasma removal on sperm quality remains controversial. Several studies reported positive effects of removing seminal plasma by centrifugation [39, 120, 121], while another found detrimental effects [122]. Sperm concentration is also critical. Post-thaw sperm quality was superior in canine samples frozen with 200 × 10^6^ or 400 × 10^6^ spermatozoa/mL compared with lower (50 × 10^6^ or 100 × 10^6^) concentrations [119]. Freezing semen at a concentration of 200 × 10^6^ spermatozoa/mL in 0.5 mL straws is also practical, as each straw contains ∼100 × 10^6^ spermatozoa, facilitating dose adjustment at insemination.

### Thawing

During thawing, spermatozoa again, must endure a lethal temperature zone. Thawing may be more harmful than freezing. Slow warming will allow time for small intracellular ice crystals to aggregate to form larger crystals. Rapid warming on the other hand will instead cause the ice to melt [26], but is associated with osmotic stress [26, 29]. Thawing is typically performed in a water bath. Pellets are usually thawed by emptying the cryovial into a whirl pack, kept in a water bath at 37–38 °C. Usually a thawing extender, or a small amount of saline is added to the whirl pack before adding the pellets. Straws are submerged in a water bath for a defined time, and then wiped dry, as water contamination is highly detrimental. Thawing rate depends on straw size and water temperature. At 37–38 °C, prolonged immersion does not risk overheating, but at ≥ 70 °C strict timing is essential. In the dog, thawing 0.5 mL straws at 70 °C for 8 s (s) produced superior results compared with 37–38 °C for 15–120 s [30, 32, 33]. In cats, thawing mini straws at 70 °C for 6 s vs. 37 °C for 15 s, yielded no difference [123], although another study reported significantly fewer acrosome reacted spermatozoa after thawing at 60 °C for 5 s, compared with 37 °C for 1 min [124]. Post-thaw dilution can reduce concentrations of toxic components (e.g., glycerol, Equex STM paste). In both species, dilution improved motility, membrane integrity, and acrosome integrity [30, 123]. However, washing feline sperm post-thaw by centrifugation reduced motility during incubation, likely due to sublethal centrifugation damage [125].

### Storage of cryopreserved semen

Frozen semen is stored in insulated cryogenic tanks filled with LN_2_. Continuous LN_2_, evaporation requires regular filling and supervision. Sperm remain safe below − 130 °C, but repeated lifting of canisters to the tank neck causes harmful temperature fluctuations [126]. Temperature will fluctuate more quickly in 0.25 mL than in 0.5 mL straws due to a higher surface-to-volume ratio [126]. Repeated cycles of elevating the straws will cause a cumulative stress [127]. Canisters should therefore not be lifted higher than necessary for handling. Canes should be held in the neck of the tank for as short period as possible. Straws should only be handled with precooled forceps and not with fingers to avoid temperature fluctuations [128]. Low LN_2_ levels increase temperature fluctuations in the tank neck, exacerbating the risk of suboptimal straw temperatures [127].

### Freezing semen that has been shipped chilled

Because relatively few clinics offer cryopreservation, it may be practical to collect semen at a local clinic, ship it chilled, and freeze it at a specialized centre [24]. This prolongs equilibration time, which can compromise semen quality. While canine semen can remain viable for several days at 4 °C, deterioration occurs over time [129]. Some studies found no adverse effects of storing semen for 24–48 h before freezing [24, 130, 131], whereas others reported reduced quality beyond 45 h [39]. The role of glycerol in cooling extenders during shipment is unclear. One study showed improved outcomes when semen was cooled in TEY + 3% glycerol and then diluted with 7% glycerol before freezing, compared with TEY without glycerol followed by 10% glycerol [24]. Other studies, however, found no difference [130]. Semen quality remained similar until day seven for semen kept at 4 °C in TEY, or TEY-3% glycerol extender (Uppsala Equex II/1), whereafter it was significantly lower in the extender with glycerol. When semen was kept at 4 °C in a TEY extender with 5% glycerol and 0.5% Equex STM paste, sperm motility declined even faster [129]. This indicates that glycerol and Equex STM paste probably have negative effects on sperm quality during long-term storage at above zero temperatures [129]. Adding an extender with 10% glycerol just before freezing may on the other hand have negative effects because of the abrupt osmotic stress [24]. Studies on different cooling times before freezing are performed under controlled conditions, in which cooling and freezing are performed in the same laboratory. Since clinical shipments involve different laboratories for cooling and freezing, standardized extenders and dilution protocols between clinics are recommended. Practically, freezing semen within 24 h of collection appears preferable to 48 h.

## Semen doses and pregnancy results after artificial insemination with frozen thawed semen

Considering that a single ejaculate typically yields only 1–4 insemination doses in the dog, compared to hundreds in the bull, data on the minimum number of spermatozoa required for successful fertilization are understandably limited. In the canine, an insemination dose with at least 150 × 10^6^ live spermatozoa is often recommended [36]. Semen doses with fewer than 100 × 10^6^ progressively motile normal spermatozoa (PMNS) resulted in significantly lower pregnancy results compared to doses with 100-150 × 10^6^ PMNS [132]. Likewise, AI doses with more than 200 × 10^6^ progressively motile sperm were significantly more likely to result in whelping compared with doses with fewer than 75-100 × 10^6^ progressively motile spermatozoa. When AI is properly timed in relation to ovulation and semen deposited with transcervical intrauterine AI, pregnancy rates of 68–75% have been reported in large clinical datasets (*n* = 352–665) [2, 132, 133]. In the cat, no large clinical datasets on AI outcomes are currently available. Transcervical AI using 20 × 10^6^ frozen-thawed spermatozoa has yielded a pregnancy rate of 41.7% (5/12) [134]. The large variation in feline semen quality, and the low sperm count per ejaculate make prediction of expected pregnancy rates more uncertain compared with the dog. Surgical intrauterine AI has been described in both dogs and cats. However, this procedure is now often considered unethical and is prohibited in some countries [135, 136].

## Alternatives to conventional semen freezing

While rapid freezing can cause lethal intracellular ice formation, ultrarapid freezing, or sperm vitrification, induces a glass-like solidification of water without ice-crystal formation. Sperm vitrification is considered easier to accomplish than traditional semen freezing, particularly under field conditions [137]. The method typically involves the use of high concentrations of penetrating CPAs and a gradual reduction of CPA-concentration during thawing. Sperm vitrification with non-permeable CPAs, such as sucrose as an alternative to the more toxic permeable CPAs has been reported both in the dog and cat [137–140]. Another alternative to conventional semen freezing is freeze-drying. In the process of freeze-drying, water is removed from the cells and spermatozoa can be stored at ambient temperature. However, a major limitation of this technique is the complete loss of sperm motility. Therefore, ICSI is required to achieve fertilization [141]. While sperm vitrification holds potential for future clinical application, freeze-drying is unlikely to be adapted in routine clinical practice due to its need for invasive advanced biotechnology for achieving pregnancy.

## Concluding remarks

A clinically applicable cryopreservation protocol must be cost-effective, user-friendly, and maintain a high proportion of spermatozoa with preserved fertilizing ability. The cryopreservation procedure is inevitably lethal for a proportion of the spermatozoa, and the surviving population has a reduced longevity caused by sub-lethal changes of the plasma membrane. Progress in composition of extenders and freezing protocols have been made by empirical studies, although the basic Tris-extender with glycerol and egg yolk, described more than 60 years ago is still used with modifications. Comparisons between studies are complicated by methodological differences, including dilution method (volume: volume vs. concentration-based), one- vs. two-step dilution, cryoprotectant concentration, and freezing rate. There may be interactions between all these factors. Results may also depend on the sperm evaluation methods. Conclusions from studies that only evaluate subjective motility, membrane integrity and acrosome integrity, might differ from studies that evaluate more sperm quality parameters. A low number of subjects evaluated will give low power to statistical comparisons between methods. Ultimately, the most relevant endpoint is in vivo fertility, but large-scale field trials are not feasible in companion animals. Thus, protocols are primarily evaluated by in vitro parameters, whose correlation with fertility remains uncertain.

## Data Availability

Not applicable.

## Abbreviations

AFP: Antifreeze proteins
AI: Artificial insemination
AMR: Antimicrobial resistance
CPA: Cryoprotective agent
EYP: Egg yolk plasma
LDL: Low-density lipoproteins
LN_2_: Liquid nitrogen
PUFA: Polyunsaturated fatty acids
PMNS: Progressively motile normal spermatozoa
ROS: Reactive oxygen species
SDS: Sodium Dodecyl Sulphate
TEY: Tris-citrate-egg yolk extender

## Declarations

### Prior publication

Data included in this article have previously been published in the Proceedings of the 25th EVSSAR Congress - European Veterinary Society for Small Animal Reproduction, Barcelona, Spain, 27–29 June 2024.
